# Design and Evaluation of FBG-Based Tension Sensor in Laparoscope Surgical Robots

**DOI:** 10.3390/s18072067

**Published:** 2018-06-28

**Authors:** Renfeng Xue, Bingyin Ren, Jiaqing Huang, Zhiyuan Yan, Zhijiang Du

**Affiliations:** 1 State Key Laboratory of Robotics and System, Harbin Institute of Technology, Harbin 150001, China; xuerenfeng@hit.edu.cn (R.X.); 17S008080@stu.hit.edu.cn (J.H.); duzj01@hit.edu.cn (Z.D.); 2 School of mechatronics Engineering, Harbin Institute of Technology, Harbin 150001, China; renby@hit.edu.cn

**Keywords:** cable tension, force sensor, surgical robot, modeling

## Abstract

Due to the narrow space and a harsh chemical environment in the sterilization processes for the end-effector of surgical robots, it is difficult to install and integrate suitable sensors for the purpose of effective and precise force control. This paper presents an innovative tension sensor for estimation of grasping force in our laparoscope surgical robot. The proposed sensor measures the tension of cable using fiber gratings (FBGs) which are pasted in the grooves on the inclined cantilevers of the sensor. By exploiting the stain measurement characteristics of FBGs, the small deformation of the inclined cantilevers caused by the cable tension can be measured. The working principle and the sensor model are analyzed. Based on the sensor model, the dimensions of the sensor are designed and optimized. A dedicated experimental setup is established to calibrate and test the sensor. The results of experiments for estimation the grasping force validate the sensor.

## 1. Introduction

Robot-assisted minimally invasive surgery technology (RMIST) provides dexterous and precise control, filters tremor of human hands, improves surgical success ratio and alleviates surgeons’ fatigue in comparison with conventional minimally invasive surgeries. Therefore, the RMIST has draw a great deal of attention during the last two decades [1,2,3]. However, there is still a crucial problem in current surgical robotic systems which is lacking of force sensation compared to conventional surgical instruments [4]. A surgeon has to operates tissues or organs based on vision without force sensation inside the patient’s body. It is extremely dangerous as an excessive grasping force may damage the patient’s tissues and organs even threaten patient’s life [5]. An insufficient grasping force, however, cannot complete surgical operation tasks, such as suturing, knotting, stripping and so on [6]. Thus, it is significantly important to measure and control the grasping force during the surgery with RMIST.

Currently, there are two main methods for predicting and evaluating surgical operation force. One method is sensorless force estimation using some intelligent algorithms to predict force. Hwang and Lim [7] propose an interaction force estimation method using visual information based on deep learning model. However, due to the complexity of surgical scene and the real-time movement of the camera during the surgery, it is not possible to learn and predict the interaction force using such a large number of sequential and variational 2D images. Moreover, the method for predicting interaction force using visual information is not suitable for hard surface which does not deform obviously under external forces such as the surface of bones. Li and Hannaford [8] propose a Gaussian process regression for sensorless force estimation to predict gripping force, which is applied to cable-driven elongated surgical instruments. Aviles et al. [9,10] make use of a neuro-visual approach to estimate the force which recovers and inputs 3D deformable structure to a deep network based on LSTM-RNN architecture. Gessert et al. [11] propose a novel image-based force estimation method using optical coherence tomography. Tsukamoto et al. [12] and Lee et al. [13] propose a reaction torque observer to estimate the grasping force without the use of force sensors. These methods for predicting interaction forces are very novel, but they are yet immature and unreliable for actual clinical application due to the uncertainty in the force acquisition method.

The other method for predicting and evaluating surgical operation forces is to design and install physical sensors in the end-effector of surgical robots. Current commercial sensors are not suitable to be integrated in the end-effector of surgical robots due to their big size. Recently, many researchers have designed and studied sensors that can be integrated in the end-effector. Kim et al. developed four degree of freedoms (DOFs) [14], six-axis [15], five DOFs [16] force and torque sensors by adopting the capacitive transduction principle, which is applied to robot-assisted minimally invasive surgery. King et al. [17] developed a multi-element tactile feedback system with a piezoresistive sensor array mounted on the graspers of a surgical robot. Dargahi et al. [18] and Qasaimeh et al. [19] designed a polyvinylidene-fluoride-based miniaturized tactile sensor which is mounted on the surgical instrument gripper. Hong and Jo [20] designed a 2-DOF compliant forceps to measure the grasping force at the tip of a surgical instrument. Lee et al. [21] designed a multi-axial contact force sensor which can measure the normal and shear force of a surgery grasper. Lee et al. [22] present a laparoscopic grasping tool which can sense three axis Cartesian manipulation force and a single axis grasping force. Mathias et al. [23] present the FBG-based force and torque sensor with six DOFs for minimally invasive robotic system (MIRS) applications. These sensors are designed to be installed in the location of the graspers of end-effectors. However, integrating the sensor into the gripper is inappropriate due to the following limitations. Firstly, it is difficult to mount, package and shield the sensor in the end-effector in actual clinical applications. Secondly, the sensor cannot bear the harsh chemical environment and high temperature in the sterilization process. Lastly, it is not economical to use the comparatively expensive force sensors for instruments due to their limited working life about 10 times. To solve this problem, some sensors are designed to attach to instrument shaft [24,25,26], articulated joint [27], trocar [2,28] and joint actuation unit [29]. However, the sensors may provide inaccurate force information due to variations of nonlinear friction, hysteresis, inertia and gravity.

Measuring the cable force is an alternative solution to improve the accuracy of the estimated grasping force. Kaneko et al. [30] present a tension differential torque sensor by using two idler pulley and a cantilever beam incorporating strain gauges. Lotti et al. [31] and Berselli et al. [32] make use of strain gauges to measure the deformation of the mechanical structure. However, a considerable disadvantage of strain gauge-based solution is its high sensitivity to electromagnetic disturbances. To solve this problem, based on FBG’s immunity to electromagnetic disturbances, some sensors are designed and developed [33,34]. Song et al. [35] present a study on the application of a FBG sensor to measure reflected forces in MIRS environments. He et al. [36] make use of three FBG sensors integrated in the forceps for vitreoretinal surgeries. Natale et al. [37] present a torque sensor based on FBG for torque control which is applied to tendon-driven mechanisms. Palli et al. [38,39,40] designed force sensors based on commercial discrete optoelectronic components mounted on a compliant frame. Although these sensors guarantee the immunity to electromagnetic disturbances, they are difficult to install, package and integrate in the end-effector of surgical robot in a narrow space.

**Figure 1:**
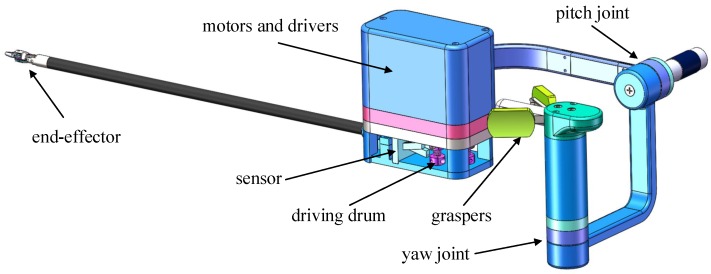
The sensor applied to investigation of force feedback and tactile sense in our prototype of laparoscope surgical robot is used to estimate and control the cable tension.

According to the model of the cable-pulley system proposed in our previous work [41], the grasping force can be inferred and analyzed from the tension of cable. Combining the strain measurement characteristics of FBGs, we designed and evaluated a force sensor integrating six cantilever beams based on FBGs for our laparoscope surgical robot shown in Figure 1. Unlike the sensors with some electronics inside, the proposed sensor is immune to electromagnetic disturbances. It can measure and estimate tensions of six cables at the same time. Meanwhile, it is mounted on the drive actuation unit side and avoids harsh chemical environment in sterilization process for the end-effector. Moreover, it is easy to install and package as the FBGs are pasted in the groove on the beams. The main contributions of this paper are modeling the sensor, optimization the dimensions of the sensor and estimating the grasping force of the end-effector based on the sensor and the model of the cable-pulley system. The sensor has considerable potential applications in many surgical robots such as da Vinci surgical robot [42].

## 2. Sensor Design

**Figure 2:**
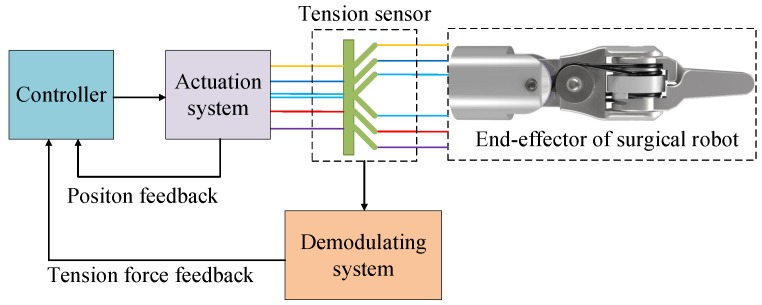
The scheme of typical cable-driven end-effector in laparoscope surgical robot.

Figure 2 shows the scheme of a typical cable-driven end-effector in laparoscope surgical robot. Six cables are used to perform the pitch and yaw motion of the end-effector. The tensions of the cables measured directly by the sensor are used as a feedback to estimate and control the grasping force and tactile force.

The design of the proposed sensor has three typical sensor specifications, enough measuring range, high sensitivity and high signal-to-noise ratio. In addition, the following specifications should be taken into account fully,

1. the structure of the sensor should avoid reducing sharply the stiffness of the transmission system which leads to an obvious hysteresis phenomenon;
2. the output of the sensor should be as linearly proportional to the input as possible;
3. the dimensions of the sensor should be minimized.

In the following section, the structure design and dimensions optimization are performed for our application.

### 2.1. Sensor Structure

**Figure 3:**
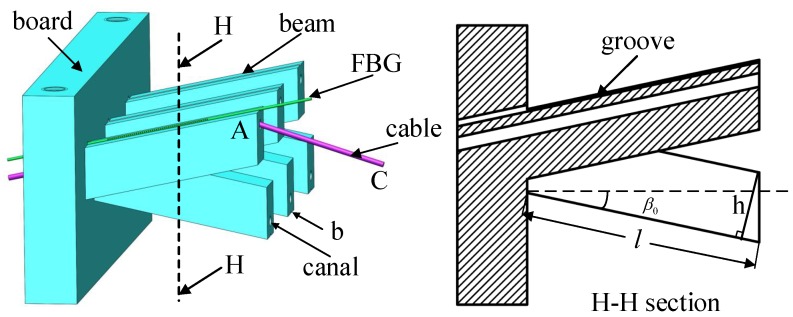
The structure of proposed sensor applied to measure cable tension in surgical robot.

The FBGs are known to have a stable and reliable wavelength response as a function of the applied strain. This characteristic inspires us to use it as a sensor in surgical robot systems to measure a small deformation of the cantilever imposed by the cable. Figure 3 shows the structure of the proposed sensor.

**Figure 4:**
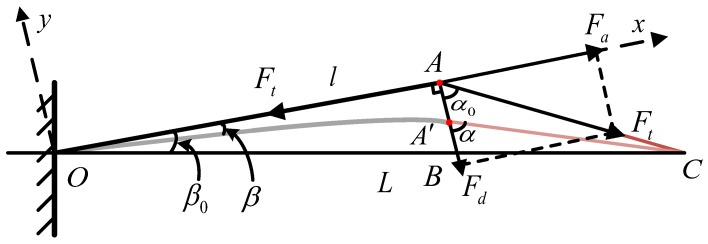
The simplified schematic that the cable tension $F_{t}$ effects on a cantilever of the sensor.

The sensor integrates six inclined cantilevers with canals. The cables are housed in the canals. The angle of the cantilever is chosen so that the cable tension produces a small deflection of the cantilever. The deflection causes a variation of strain of the beam which is measured by the FBG. The FBG is located and pasted in the groove on the surface of the cantilevers with the glue Alteco SG-12 produced by Alteco Chemical Ltd., Japan. It is assumed that the FBG is connected rigidly to the beam. Figure 4 shows a simplified schematic where the cable tension $F_{t}$ exerts on the sensor. The cable CAO passes through the cantilever AO with an initial angle $\beta_{0}$. The decomposed cable tension $F_{d}$ and $F_{a}$ cause a small deflection of the cantilever from OA to $OA^{\prime }$ with an angle change of β. To be exact, the tension $F_{d}$ and $F_{a}$ cause the bending and stretching of the beam, respectively.

The decomposed tension $F_{d}$ and $F_{a}$ can be expressed by the equations,

(1) $$F_{d}=F_{t}\cos\alpha$$

(2) $$F_{a}=F_{t}\sin\alpha$$

where α is the angle between the cable and the beam and $\alpha_{0}$ is the initial angle. Since the deflection of cantilever $AA^{\prime }$ is small, it is assumed that the direction of tension $F_{d}$ does not change. The deflection of $AA^{\prime }$ is defined as *w* which is expressed by the equaltion,

(3) $$w=\frac{F_{d}l^{3}}{3EI}$$

where *E* is the Young’s modulus of the material and *I* is cross sectional inertia moment which is expressed by the equation,

(4) $$I=\frac{bh^{3}}{12}.$$

where *h* and *b* are the height and the thickness of the beam, respectively. The geometry relationships shown in Figure 3 are expressed by the equations,

(5) $$\begin{array}{c} \left|OB\right|=\frac{l}{\cos\beta_{0}}\\ \left|AB\right|=l\tan\beta_{0}\\ \left|BC\right|=L-\left|OB\right|\\ \left|AC\right|=\sqrt{L^{2}+l^{2}-2Ll\cos\beta_{0}}\\ \left|A^{\prime }B\right|=\left|AB\right|-w\\ \left|A^{\prime }C\right|=\sqrt{w^{2}+{\left|AC\right|}^{2}-2\left|AC\right|w\cos\alpha_{0}} \end{array}.$$

where *L* is the length between the board of sensor and driving drum, *l* is the length of the cantilever and $\beta_{0}$ is the initial inclined angle of the cantilever. According to the cosine theorem, the angle between the beam and the cable is calculated by the equations,

(6) $$\cos\alpha_{0}=\frac{{\left|AB\right|}^{2}+{\left|AC\right|}^{2}-{\left|BC\right|}^{2}}{2\left|AB\right|\;\cdot \;\left|AC\right|},$$

(7) $$\cos\alpha =\frac{{\left|A^{\prime }B\right|}^{2}+{\left|A^{\prime }C\right|}^{2}-{\left|BC\right|}^{2}}{2\left|A^{\prime }B\right|\;\cdot \;\left|A^{\prime }C\right|}.$$

Substituting Equations (5)–(7) into Equation (1), the decomposed tension $F_{d}$ and $F_{a}$ can be calculated where sinα can be experessed by

$$\sin\alpha =\sqrt{1-{\left(cos\alpha \right)}^{2}}.$$

According to Equation (3), the deflection of beam *w* caused by the cable tension $F_{t}$ can be evaluated by calculating the mentioned geometry relations.

The deflection *w* leads to strain $\varepsilon_{x}$ along the surface of the beam which can be measured by FBGs. The sensor uses FBGs for their many advantages compared with conventional electrical strain gauges, such as its excellent linear characteristics, immunity to Electromagnetic Interference (EMI) and low fiber loss. FBG reflects a narrow spectral part of light is guided in the fiber core at Bragg wavelength $\lambda_{B}$, which depends on the grating period and the refractive index of the fiber. The wavelength $\lambda_{B}$ of the reflected light (Bragg wavelength) is expressed by,

(8) $$\lambda_{B}=2n_{e}\Lambda$$

where $n_{e}$ and Λ are the effective refractive index of the fiber core and the grating period, respectively. The change in the refractive index or in grating period will cause a shift $\Delta \lambda_{B}$ in the wavelength of the reflected light. The wavelength shift depends on the strain $\varepsilon_{x}$ and temperature *T* and is expressed by the equation,

(9) $$\frac{\Delta \lambda_{B}}{\lambda_{B}}=\left\{1-\frac{n_{e}^{2}}{2}\left[P_{12}-\upsilon \left(P_{11}+P_{12}\right)\right]\right\}\varepsilon_{x}+\left(\alpha +\frac{dn_{e}}{dT}\frac{1}{n_{e}}\right)\Delta T$$

where $P_{11}$ and $P_{12}$ are the photoelastic coefficient of the fiber, υ is Poisson’s ratio of the fiber, α is the coefficient of thermal expansion of the fiber material, and ΔT is the change of the temperature. It is assumed that the environment temperature is constant (i.e., ΔT=0). Therefore, the effect of temperature is ignored and the equation is rewritten as

(10) $$\frac{\Delta \lambda_{B}}{\lambda_{B}}=\left(1-\rho_{\alpha }\right)\varepsilon_{x}$$

where

$$\rho_{\alpha }=\frac{n_{e}^{2}}{2}\left[P_{12}-\upsilon \left(P_{11}+P_{12}\right)\right].$$

According to the mechanics of materials the strain $\varepsilon_{x}$ along the fiber is expressed by

(11) $$\varepsilon_{x}=\varepsilon_{1}+\varepsilon_{2}$$

where $\varepsilon_{1}$ is the strain caused by bending effect, $\varepsilon_{2}$ is the strain caused by stretching effect. The bending effect and stretching effect are mainly caused by the normal force $F_{d}$ and tangential force $F_{a}$, respectively. They can be expressed by the equations,

(12) $$\varepsilon_{1}=\frac{M_{x}}{EI}\;\cdot \;\frac{h}{2}$$

(13) $$\varepsilon_{2}=\frac{F_{a}-F_{t}}{EA}$$

where,

$$M_{x}=F_{d}l/2$$

represents bending moment of the beam considering the strain measured by the FBG is at the location l/2, parameter *A* represents the effective cross section area which is equal to *b* times *h*. Combining Equations (1), (2), (12) and (13), Equation (11) can be rewritten as

(14) $$\varepsilon_{x}=\frac{F_{t}\left\{3l\cos\alpha -h\left(1-\sin\alpha \right)\right\}}{Ebh^{2}}$$

It is seen that if 3lcosα−h(1−sinα) in Equation (14) is approximately constant in quasi-linear domain (small deformations), the relationship between the strain of beam and the cable tension is approximately linear. A linear approximation in Equation (14) is expressed by

(15) $$\varepsilon_{x}≃K_{l}F_{t}.$$

Substitute Equation (15) into Equation (10), the relationship between the wavelength shift $\Delta \lambda_{B}$ and the cable tension $F_{t}$ is expressed by

(16) $$\frac{\Delta \lambda_{B}}{\lambda_{B}}=K_{l}\left(1-\rho_{\alpha }\right)F_{t}.$$

Therefore, the tension of cable can evaluated by the equation

(17) $$F_{t}=\frac{\Delta \lambda_{B}}{K_{l}\left(1-\rho_{\alpha }\right)\lambda_{B}}$$

In the following section, the analysis mentioned in this section will be used to design and optimize initial parameters of the sensor for our application of surgical robot. The specifications mentioned are taken into account fully. The sensor should not reduce sharply the stiffness of the transmission system. It should have a good linearity and sensitivity. The dimensions of the sensor should be as small as possible for the narrow space of surgical robot.

### 2.2. Parameters of Sensor Optimization

The main structure parameters of the sensor are length *l*, width *b*, height *h* and initial inclined angle $\beta_{0}$ shown in Figure 3. The dimensions of the board integrating cantilevers depend on the location in the surgical robot shown in Figure 1. The length, width and height of the board are 40 mm, 5 mm and 28 mm, respectively. The radius of canal is equal to 1 mm, slightly larger than the radius of the cable 0.45 mm. Small and large radius of canals lead to a considerable friction loss and a reduced stiffness of the beam, respectively. The depth of groove is 0.25 mm and the diameter of FBG is 0.125 mm. The groove protects effectively the fiber grating and prevents the sideways slip of the fiber.

To avoid lateral force on the beam acted by the cable, the distance of a pair of canals is equal to the radius of the driving drum 5.5 mm. Considering the radius of the cable, the value of the width of beam is selected as *b* = 4 mm. The vertical distance between two canals on the board throughout a pair of beams is equal to a screw pitch of driving drum 0.6 mm in order to minimize the influence of friction loss. Considering the length between the board and driving drum *L* = 27 mm, the length of beam *l* is selected between 14 mm and 17 mm (*l* = 14∼17 mm). Low value of *l* needs high value of *h* to ensure that the deflection *w* has a small variation range which guarantees validity of the sensor model and good linearity between the cable tension and the wavelength shift in Equation (14). In fact, we need a large value of *h* to prevent a plastic deformation caused by a large force and avoid a zero point shift relating to the deformation. However, a large value of *h* will sacrifice some sensitivity.

The initial inclined angle $\beta_{0}$ is limited in a narrow space of the surgical robot considering the specification 3. The initial inclined angle $\beta_{0}$ is selected from $7^{\circ }$ to $12^{\circ }$ ($\beta_{0}=7^{\circ }$∼$12^{\circ }$). Large value of $\beta_{0}$ leads to a large friction loss between the cable and the beam and a low stiffness of the sensor in the direction of the transmission force. Small value of $\beta_{0}$ reduces the sensitivity of the sensor.

Before the dimensions of the structure are optimized, the choice of material should be determined. Metal material is inappropriate for the sensor as the tension of cable is difficult to deform the beam because of its high stiffness. Meanwhile a small strain of beams introduces a large measurement error. Shape memory alloys (SMA) have a superelastic property. It can provide a large strain of beams and a good anti-fatigue property. However, it reduces the stiffness of the transmission system seriously which is against to specification 1. Moreover, the elastic module of SMA is changed with strain of current and previous moment [43]. Plastic is a suitable choice due to its easy processing and low cost. Meanwhile, it can provide a sufficient deformation while maintain the stiffness of the transmission system.

To meet specification 2, a value *w* is assumed sufficiently small so that the sensor remains in a quasi-linear deformation range. It needs a small variation of inclined angle β (less than 10 % $\beta_{0}$). Based on the geometrical relationship in Figure 4, the deformation value *w* is defined as 0.3 mm assuming that the length of beam *l* and the initial inclined angle $\beta_{0}$ are 17 mm and $12^{\circ }$, respectively. In the variation range of *w* (w=0∼0.3 mm), based on Equations (1) and (2) the needed cable tension $F_{t}$ acting on the beam can be calculated by

(18) $$F_{t}=\frac{3EIw}{l^{3}\cos\alpha }.$$

To optimize the structure parameters of the sensor *l*, $\beta_{0}$ and *h*, an evaluation function of linearity is defined by

(19) $$\delta_{\max}=\max\left(\frac{\varepsilon_{x}-\varepsilon_{l}}{\max\{\varepsilon_{l}\}}\times 100\%\right)$$

where the desired cable tension with pure linearity $F_{l}$ is expressed by

$$\varepsilon_{l}=\frac{F_{t}\max\left\{\varepsilon \right\}}{70}$$

in the case of the maximum value of cable tension (70 N) and beam deflection (0.3 mm). It is possible to choose suitable $\beta_{0}$ and *l* to minimize the evaluation function of linearity by solving the following problem,

(20) $$\min\delta_{\max}\left(\beta_{0},l\right).$$

**Figure 5:**
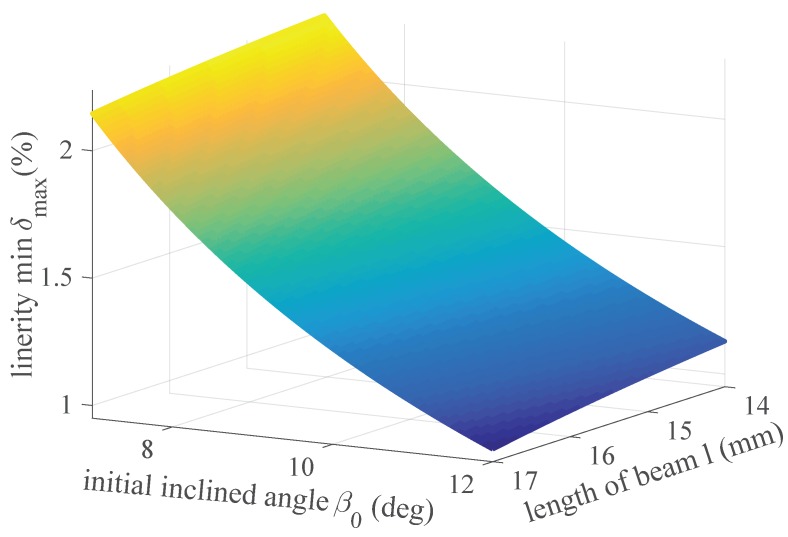
Analysis for optimal solution of evaluation function of linearity with the variation of the length of beam *l* and initial inclined angle $\beta_{0}$.

According to Equation (18), the evaluation function of linearity is shown in Figure 5. It is seen that when the values of *l* and $\beta_{0}$ are maximum values (l= 17 mm, $\beta_{0}=12^{\circ }$) the linearity has an optimal solution 0.9986 %. In the case of the optimal solution, the height of beam *h* is solved by Equations (3) and (4) and selected *h* = 6 mm.

**Figure 6:**
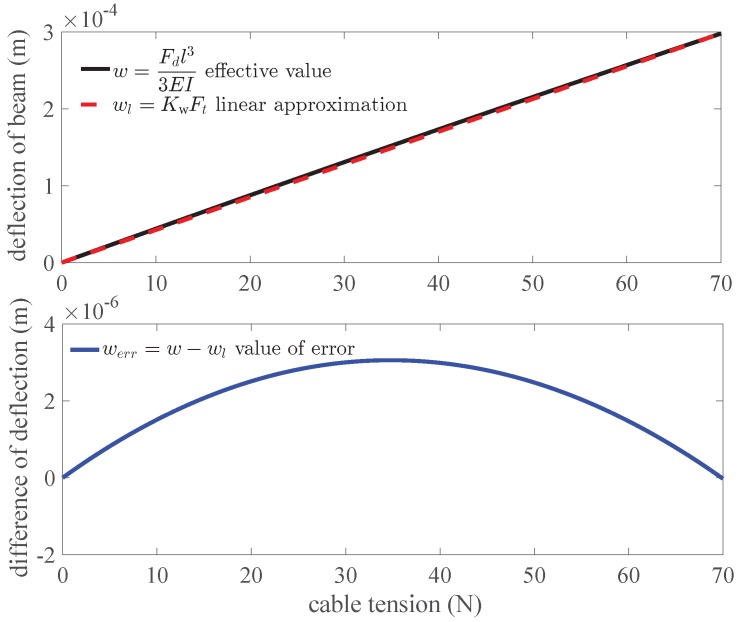
The deflection of beam *w* changes with the cable tension $F_{t}$ in Equation (3) with a linear approximation $w_{l}$ in the top panel and the difference of them $w_{err}$ in the bottom panel.

**Figure 7:**
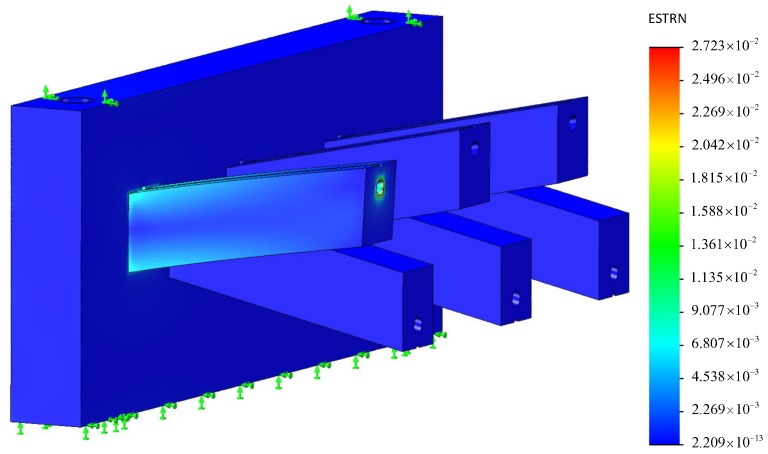
The FEM analysis of one beam of the sensor in the maximum value of the cable tension 70 N.

Figure 6 shows that the deflection of beam *w* changes with the cable tension $F_{t}$ in the Equation (3) with a linear approximation $w_{l}$ in the top panel and the difference of them $w_{err}$ in the bottom panel. The maximum value of the deviation is 3.055 μm. The deflection of beam *w* has a good linearity 1.018% which meets the specification 2. Figure 7 shows FEM analysis of one beam of the sensor in the maximum value of the cable tension 70 N. The material of simulation is selected as Polyamide (PA) with elastic module of 2.62 GPa. It is close to the material photopolymer of the sensor. A tension of 70 N is exerted on the edge of the canal. The board of the sensor is fixed. The mesh is generated automatically by the software SolidWorks Simulation. The results of analysis show that the maximum value of equivalent strain on the beam computed by FEM analysis is 0.0272.

**Figure 8:**
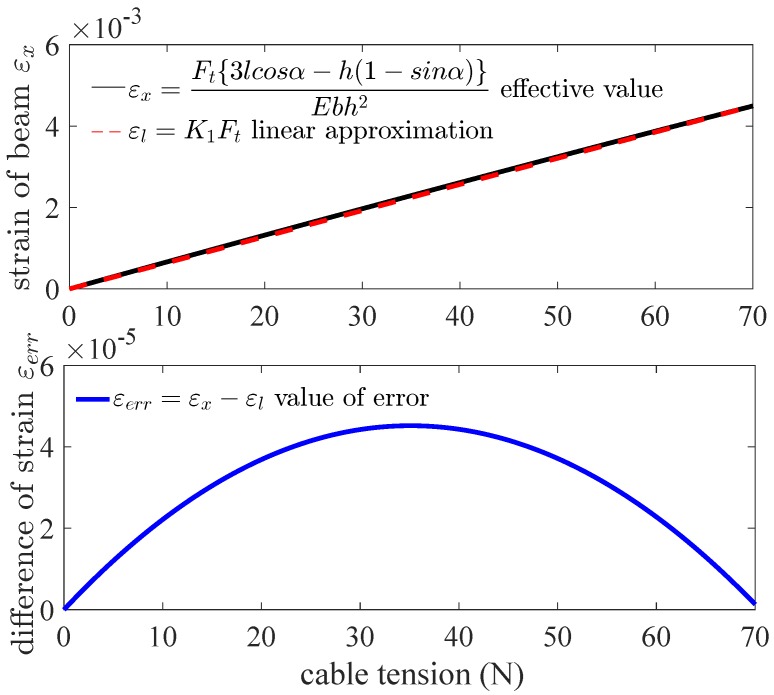
The strain of beam $\varepsilon_{x}$ changes with the cable tension $F_{t}$ in Equation (14) with a linear approximation $\varepsilon_{l}$ in the top panel and the difference of them $\varepsilon_{err}$ in the bottom panel.

Figure 8 shows the strain of beam $\varepsilon_{x}$ changes with the cable tension $F_{t}$ in Equation (15) with a linear approximation $\varepsilon_{l}$ in the top panel and the difference of them $\varepsilon_{err}$ in the bottom panel. The relation between the strain of beam and the cable tension is a linear approximation in quasi-linear domain on the basis of Equation (15) although there are trigonometric functions in the equation. The maximum value of the difference of strain $\varepsilon_{err}$ is $4.52\times 10^{-5}$.

## 3. Sensor Calibration and Testing

**Figure 9:**
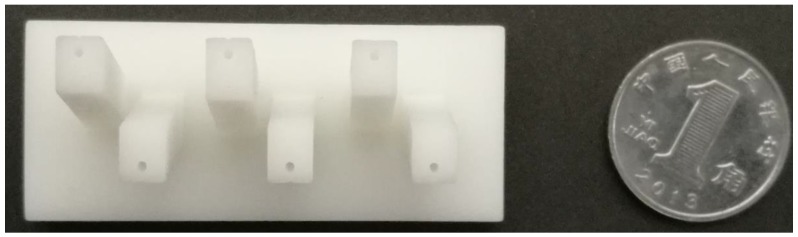
The prototype of the sensor manufactured by means of rapid protoying technology.

The sensor mentioned is manufactured by rapid prototyping in photopolymer C-UV 9400 which is a polymer that changes its properties when exposed to light, often in the ultraviolet or visible region of the electromagnetic spectrum. It is manufactured by Xuansheng Model Co., Ltd., Dongguan, China. The prototype of the proposed sensor is shown in Figure 9. The details about design of sensor are illustrated in Figure 3.

### 3.1. Calibration of the Sensor

**Figure 10:**
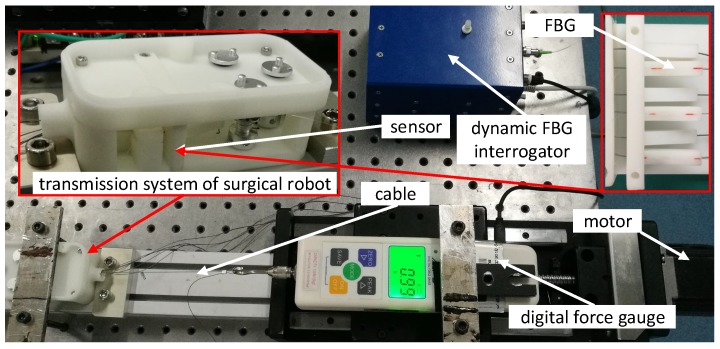
An experimental setup to calibrate the proposed sensor.

Figure 10 shows an experimental setup for calibration of the sensor. The prototype of the sensor is mounted in the transmission system of the surgical robot. The cable is tungsten steel wire with 0.45 mm diameter which is a commercial product of medical application made by DMC Corporation, Osaka, Japan. The cable is connected to the digital force gauge AIPU HF-10, produced by FuZhou AIPU Instruments Co., Ltd., FuZhou, China, which is fixed on the mobile platform. The platform is dragged along the slide guide by a motor. The capacity and resolution of the digital force gauge are 100 N and 0.01 N, respectively. The optical signal of FBG is interrogated by the dynamic FBG interrogator SartScan produced by Smart Fibres Ltd., Bracknell, UK, which can capture accurately the spectrum from 1528 nm to 1568 nm with a frequency of 2.5 kHz.

**Figure 11:**
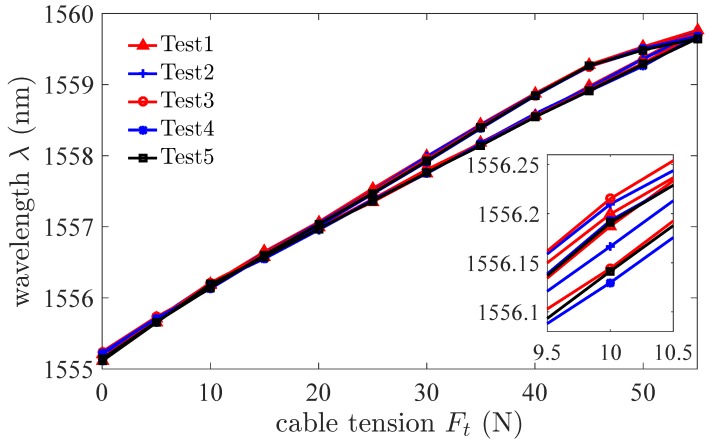
The wavelength λ changes with the cable tension $F_{t}$ in the direction of increasing input and in the direction of decreasing input.

**Figure 12:**
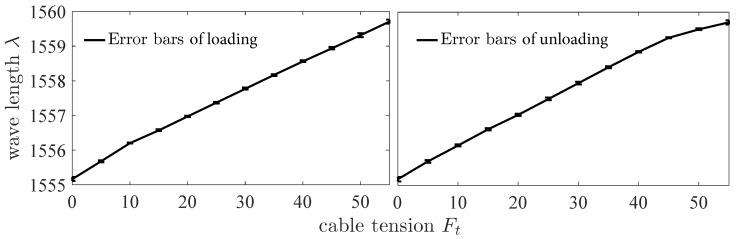
The error bars of wavelength λ changes with the cable tension $F_{t}$ in the direction of increasing input. The error bars represent standard deviation with 5 repeats.

The cable tension is increased and decreased by moving the platform which is driven by the motor. The change of the cable tension causes the change of the beam strain. The strain is measured by FBG which is interrogated by the dynamic interrogator. Figure 11 shows that the wavelength λ changes with the cable tension $F_{t}$ in the directions of loading and unloading. The results of experiments show that the sensor has a hysteresis characteristic. The hysteresis characteristic is the difference in the sensor output response during loading and unloading at the same force. The maximal value of hysteresis error is 7.9%. The repeatability of the sensor in the direction of loading is ±2.71%. The standard deviation is 0.1158 nm. Figure 12 shows the error bars of wavelength λ changes with the cable tension $F_{t}$ in the directions of loading and unloading. The error bars represent standard deviation with 5 repeats. The results of experiments show that the linearity of the sensor is ±5.57%. The sensitivity of the sensor is 0.0838 nm/N. As the background noise is ±5 pm, the resolution of the sensor calculated is 0.14 N according to Equation (17). The signal to noise ratio of the sensor is 57.35 dB. The maximum load of the sensor is 68.6 N for the maximum change of the wavelength is 5 nm. In the surgery process, for a needle, the grasping force of end-effector of Da Vinci surgical robot is about 10 N to 16 N. As we use cable-pulley system to transmit the force and displacement, there is a large friction loss. According to our previous experiments, the value of tension in driving side is up to 45 N or even higher to provide a grasping force of 15 N. Thus, the maximum load of the sensor 68.6 N is sufficient for surgical applications.

### 3.2. Testing of the Sensor

**Figure 13:**
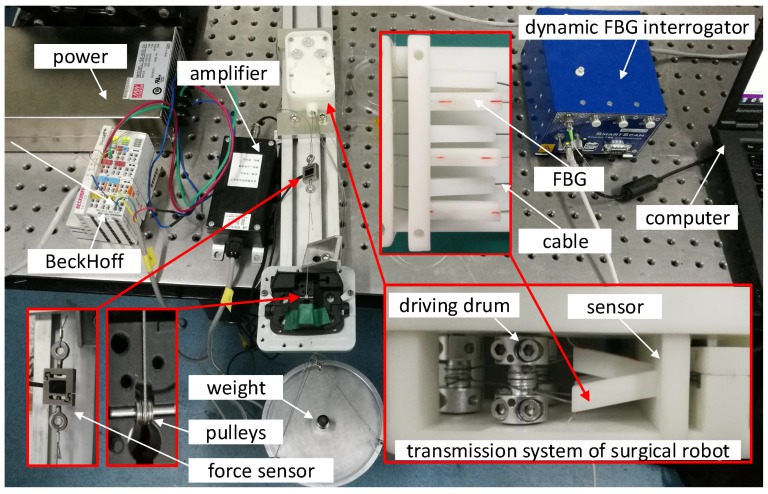
Experiments to investigate and test the proposed sensor mounted in the transmission system of surgical robot.

To validate and test the sensor, a dedicated experimental setup is established shown in the Figure 13. A force sensor (JLBS-MD), which measures tension of cable, is used to compare with the result of measurement of the proposed sensor. The tension signal of force sensor is amplified by the BSQ-2 voltage amplifier and captured by the BeckHoff controller.

The tension of the cable is increased continually by the weight. The dynamic FBG interrogator captures and records the wavelength shift which changes with strain of the beam. The initial wavelength $\lambda_{B}$ is 1555.2671 nm. The parameter $\rho_{\alpha }$ is 0.216 which is calculated according to Equation (10) where the photoelastic coefficients of fiber $P_{11}$ and $P_{12}$ are 0.121 and 0.270, respectively, Poisson’s ratio of the fiber υ is 0.17, the effective refractive index of fiber $n_{e}$ is 1.456.

**Figure 14:**
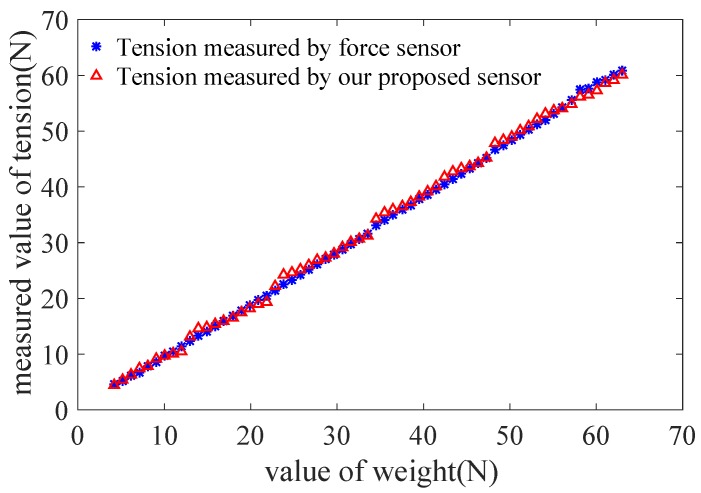
A comparison of tension measured by force sensor (JLBS-MD) and our proposed sensor.

The tension is measured and calculated based on Equation (17). Figure 14 shows a comparison of value of tension measured by force sensor (JLBS-MD) and our proposed sensor. The values of tension measured are fitted by the least square method. The values of square of the correlation coefficient of them are 0.9995 and 0.9983, respectively. Based on above results of experiments, although the proposed sensor is slightly inferior to the force sensor (JLBS-MD), it has already met our requirements in application of surgical robot mentioned.

## 4. Estimation of the Grasping Force Based on the Sensor

**Figure 15:**
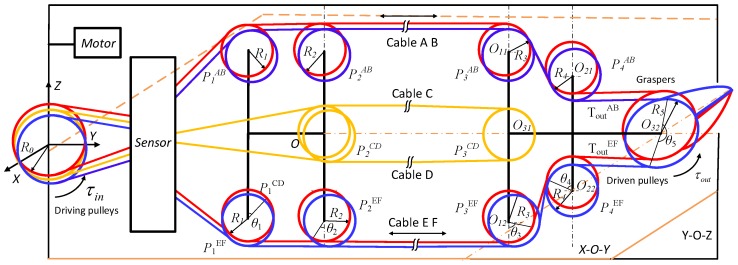
The structure of cable-pulley system in the end-effector. Six cables are used to control the end-effector with two DOFs. The sensor is used to measure the tensions of cables A∼F.

In this section, the grasping force of the end-effector is estimated based on the sensor. Figure 15 shows the structure of the cable-pulley system in the end-effector. The end-effector with 2-DOF, pitch and yaw motion, is controlled by six cables. Cables A & E and B & F are used to control yaw motion of the right and left graspers, respectively. Cables C & D are used to control pitch motion of graspers. The beams of sensor are used to measure the tensions of the cables A∼F, respectively.

In our previous works [41], we build the model of cable-pulley system for the end-effector in surgical robot. The output torque of the end-effector is expressed by

(21) $$\tau_{\mathrm{out}}\left(t\right)=\left\{\begin{array}{c} \frac{R_{5}}{R_{0}}\;\cdot \;\frac{MG+NF}{F+G}\;\cdot \;\tau_{in}+\frac{\left(M-N\right)R_{5}\lambda }{F+G}\;\;\;\;\;\;\;\;\;\;\;\;\left(\dot{\xi }>0\right)\;\;\\ \frac{R_{5}}{R_{0}}\;\cdot \;\frac{M^{*}G+N^{*}F}{F+G}\;\cdot \;\tau_{in}-\frac{\left(M^{*}-N^{*}\right)R_{5}\lambda }{F+G}\;\;\;\;\;\;\left(\dot{\xi }<0\right)\;\;\;\;\;\;\;\;\;\\ \tau_{\mathrm{out}}\left(t^{-1}\right)\;\;\;\;\;\;\;\;\;\;\;\;\;\;\;\;\;\;\;\;\;\;\;\;\;\;\;\;\;\;\;\;\;\;\;\;\;\;\;\;\;\;\;\;other \end{array}\right.$$

where, $\dot{\xi }$ represents the motion direction of the graspers; $\dot{\xi }>0$ represents anticlockwise and $\dot{\xi }<0$ represents clockwise; $M,N,F,G,M^{*},N^{*}$ are the parameters of the cable-pulley system which are expressed by

$$M=\prod_{1}^{4}\left[1/K\left(\theta_{i}\right)\right],\;N=\sigma \prod_{1}^{4}K(\theta_{i}),$$

$$M^{*}=\gamma \prod_{1}^{4}[1/K\left(\theta_{i}\right)],\;N^{*}=\prod_{1}^{4}\left[K\left(\theta_{i}\right)\right],$$

$$F=\sum_{i=2}^{5}\left\{\left.(\prod_{j=1}^{i-1}\frac{1}{K(\theta_{j})})\left(R_{i}\int_{0}^{\theta_{i}}\frac{1}{K(\varphi )}d\varphi +d_{i}\right)\right\}\right.+d_{1},$$

$$G=\sum_{i=2}^{5}\left.\left\{(\prod_{j=1}^{i-1}K(\theta_{j})\right.)\left(R_{i}\int_{0}^{\theta_{i}}K(\varphi )d\varphi +d_{i}\right)\right\}+d_{1},$$

$R_{i}(i=1$∼5) is the radius of pulleys; $d_{i}(i=1$∼5) is the length of the cable between adjacent pulleys; $K(\theta_{i})$ represents the ratio of incoming tension to outgoing tension; the input torque $\tau_{in}$ is calculated according to the tensions of cables. The grasping force $f_{Grasp}$ is estimated by

(22) $$f_{Grasp}\left(t\right)=\tau_{out}\left(t\right)/\left(\frac{l_{g}}{2}\right),$$

where $l_{g}$ is the length of the grasper.

**Figure 16:**
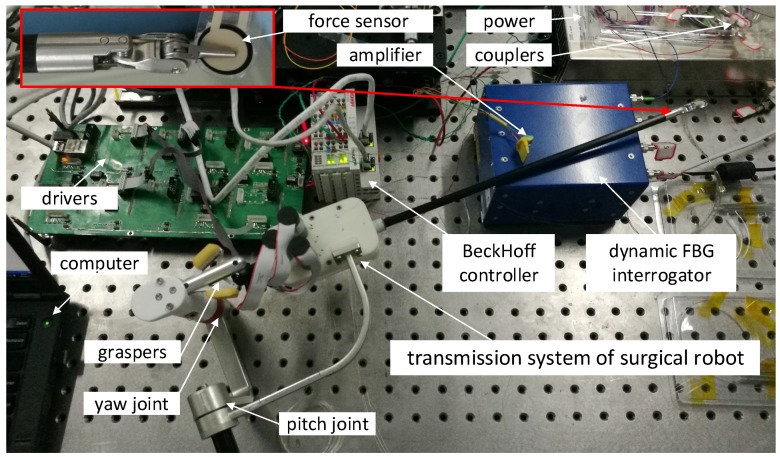
An experiment setup to estimate the grasping force based on the proposed sensor and the structure of cable-pulley system in the end-effector.

Figure 16 shows an experimental setup to estimate the grasping force based on the proposed sensor. The sensor is installed in the transmission system of the surgical robot. The tensions of the six cables are measured by the sensor. Two couplers are used to connect the FBGs as the dynamic FBG interrogator only has four channels. The end-effector is driven by three motors which transmit the force by cable-pulley transmission system. The grasping force of graspers is measured by Flexiforce sensor made by TeKscan Corporation, San Jose, CA, USA. The force signal is amplified, and acquired by BeckHoff controller module.

**Figure 17:**
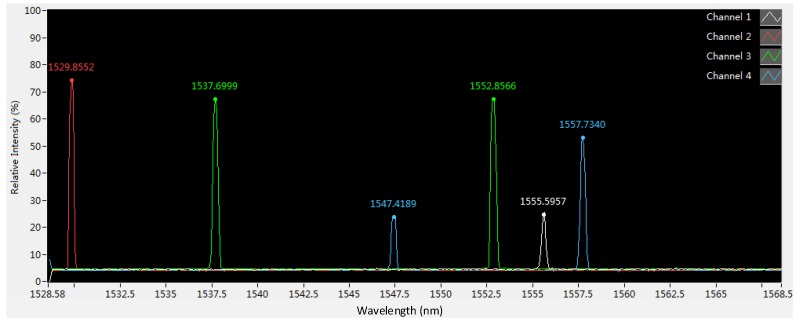
The spectrum of six FBG integrated in the sensor.

Figure 17 shows six spectrums of six FBGs integrated in the sensor. The values of the relative intensity of 4 FBGs are 60∼80%. However, two values of the relative intensity are about 25%. It shows that the power loss of the two FBGs is relatively large due to the fiber bending outside the sensor. If the value of the relative intensity is less than 20%, the interrogator cannot capture the spectrum of FBG. Therefore, the fiber bending outside the cantilevers should be as small as possible. The temporal resolution of the measurement is 2 ms as the dynamic FBG interrogator works at 500 Hz. The interrogator can measure the six spectrums simultaneously.

**Figure 18:**
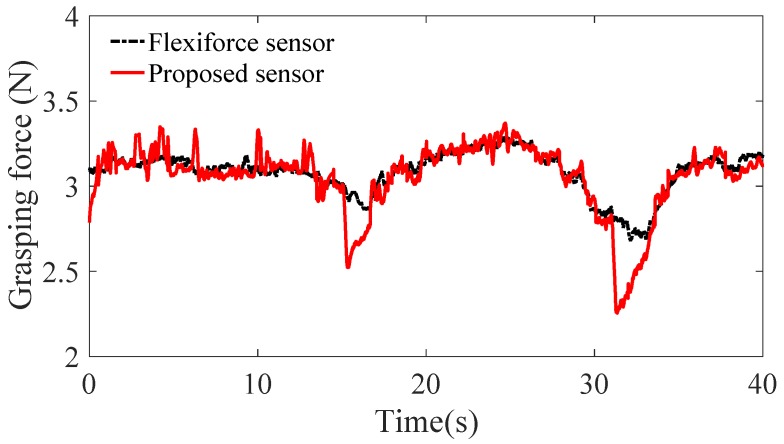
A comparison of grasping force measured by Flexiforce sensor and our proposed sensor.

The graspers of end-effector are controlled by the handle. Based on the model mentioned, the tensions of six cables measured and calculated are used to estimate the grasping force. Figure 18 shows a comparison of grasping force measured by Flexiforce sensor and our proposed sensor. There are two wave troughs at the moment 16 s and 31 s for the motion direction of graspers are changed. When the motor change the motion direction, the tension of one cable decreases sharply and the other increases sharply. Thus, the input torque has a peak that leads to a peak of estimation for grasping force. The maximum value of difference between the two sensors measured is 0.5 N at the moment of direction change.

## 5. Discussion

A tension sensor based on FBG in laparoscope surgical robot is designed and evaluated. Considering application in narrow space of surgical robot, the dimensions of sensor should be minimized. Meanwhile the specifications 1 and 2 mentioned in Section 2 should be taken into account fully. For this problem, the dimensions of the sensor are designed and optimized. Figure 5 shows the result of optimization. It is seen that choosing large values of both length of beam *l* and initial inclined angle $\beta_{0}$ has a good linearity of the sensor. However, large values of *l* and $\beta_{0}$ lead to a large friction loss and a low stiffness of the transmission system. A compromised choice is that the length *l* and the initial inclined angle $\beta_{0}$ of beam are 15 mm and $12^{\circ }$, respectively. Based on the choices of *l* and $\beta_{0}$, the thickness of beam *h* is selected 6 mm with respect to Equations (3) and (4) where the width of beam *b* is 4mm considering the radius of driving drum 5.5 mm to avoid lateral force on the beam. A large value of *h* will sacrifice some sensitivity of the sensor, but it prevents a plastic deformation caused by a large force and avoids a zero point shift of the deformation.

Based on above selected dimension parameters of the sensor, the linear relation between the input and output of the sensor is simulated and analyzed in quasi-linear domain. It meets the specification 2. As shown in Figure 6, the deflection of beam *w* is proportional to the normal force $F_{d}$ approximatively in the case of small deformation of the beam. The strain of beam $\varepsilon_{x}$ consists of two parts, $\varepsilon_{1}$ caused by bending effect of normal force $F_{d}$ and $\varepsilon_{2}$ caused by stretching effect of tangential force $F_{a}$ according to Equation (11). If the relation of the strain ε and the cable tension $F_{t}$ is quasi-linear, the variation of output wavelength Δλ is proportional to the input tension $F_{t}$ according to Equation (10). Figure 8 validates the linear approximation of Equation (15). The sensor is tested and calibrated as shown in Figure 10. The results of experiments are shown in the Figure 11 and Figure 12. There are two inflection points at 10 N in direction of loading and at 45 N in the direction of unloading. This is mainly caused by the friction between the cable and beams. The friction changes the sensitivity of the sensor. Then the tension measured by our proposed sensor is compared with the tension measured by the force sensor (JLBS-MD) shown in Figure 14. Both of them show a good linear relationship with the external load. The tension measured by our proposed sensor has several steps which are mainly caused by disturbances and hysteresis errors of the sensor in the process of applying weights. The tensions, which are measured by the sensor, are used to calculated the grasping force based on the model of the cable pulley system. Figure 18 shows a comparison of grasping force measured by the sensor and Flexiforce sensor. The results of experiments show that the sensor can be used to estimate the grasping force well. The maximum value of errors is about 0.5 N. There is an unsatisfied estimation at the moment when the motor changes the motion direction. It is mainly caused by the accuracy of the model.

In this paper, we neglect the effect of wearing and tearing between the cable and the canal. With regard to wearing and tearing between the cable and the canal, 100 repeated tensile experiments are performed. A weight of 30 N is hung at the end of the cable and pulled repeatedly. The results of experiments show that the edge of the canal is wear lightly and the canal is enlarged about 0.4 mm. Thus, the accuracy of the sensor will become low with the use of the sensor. We also neglect the effect of the fiber delamination in this paper where it is assumed that the FBG is connected rigidly to the beam. Although the thickness of the glue is controlled as small as possible during the adhesive process by adsorbing excessive glue, the delamination of the fiber still affects the measurement results. As the background noise of the FBG dynamic interrogator is ±5 pm, in theory, the minimum value of force that can be sensed is 0.14 N. However, both the hysteresis characteristics of the sensor and the friction between the cable and beams affect the resolution of the sensor.

## 6. Conclusions

In this paper, a tension sensor based on FBG in laparoscope surgical robot is designed and evaluated. The sensor integrating inclined cantilevers is modeled. Based on the model, the dimensions of the sensor are designed and optimized taking into account of the specifications in our application of surgical robot. For the optimized dimensions, the linear relation between the strain of beam and cable tension in quasi-linear domain is simulated and validated. A dedicated experiment setup is established to calibrate and test the sensor. The results of experiments show that the sensitivity, linearity, hysteresis error, repeatability, standard deviation, resolution and maximum load of the sensor are 0.0838 nm/N, ±5.57%, 7.9%, ±2.71%, 0.1158 nm, 0.14 N and 68.6 N, respectively. A comparison of tension measured by the force sensor (JLBS-MD) and our proposed sensor. The results show that the proposed sensor has a good performance as JLBS-MD. An experiment setup is established to estimate the grasping force of our surgical robot. The results of experiments show the sensor can be used to estimate the grasping force.

The future work is to test and interrogate dynamic tension signals of six cables, then apply the signals to control the surgical robot with force feedback. In addition, packaging the FBG of sensor and integrating the dynamic FBG interrogator will be studied and thought carefully.
